# Small molecule inhibitors of osteoarthritis: Current development and future perspective

**DOI:** 10.3389/fphys.2023.1156913

**Published:** 2023-04-07

**Authors:** Dan Liu, Xingxing Li, Lin Zhang, Bin Hu, Sang Hu, Xiao Zhang, Jing Hu

**Affiliations:** ^1^ Department of Pharmacy, The First Affiliated Hospital of Army Medical University (Third Military Medical University), Chongqing, China; ^2^ Institute of Pathology, The First Affiliated Hospital of Army Medical University (Third Military Medical University), Chongqing, China; ^3^ Chongqing Institute of Advanced Pathology, Jinfeng Laboratory, Chongqing, China

**Keywords:** osteoarthritis, cartilage, pharmacology, small molecule inhibitor, subchondral bone

## Abstract

Osteoarthritis (OA) is one of the common degenerative joint diseases in clinic. It mainly damages articular cartilage, causing pain, swelling and stiffness around joints, and is the main cause of disability of the elderly. Due to the unclear pathogenesis of osteoarthritis and the poor self-healing ability of articular cartilage, the treatment options for this disease are limited. At present, NSAIDs, Glucocorticoid and Duloxetine are the most commonly used treatment choice for osteoarthritis. Although it is somewhat effective, the adverse reactions are frequent and serious. The development of safer and more effective anti-osteoarthritis drugs is essential and urgent. This review summarizes recent advances in the pharmacological treatment of OA, focusing on small molecule inhibitors targeting cartilage remodeling in osteoarthritis as well as the research idea of reducing adverse effects by optimizing the dosage form of traditional drugs for the treatment of osteoarthritis. It should provide a reference for exploration of new potential treatment options.

## 1 Introduction

**FIGURE 1:**
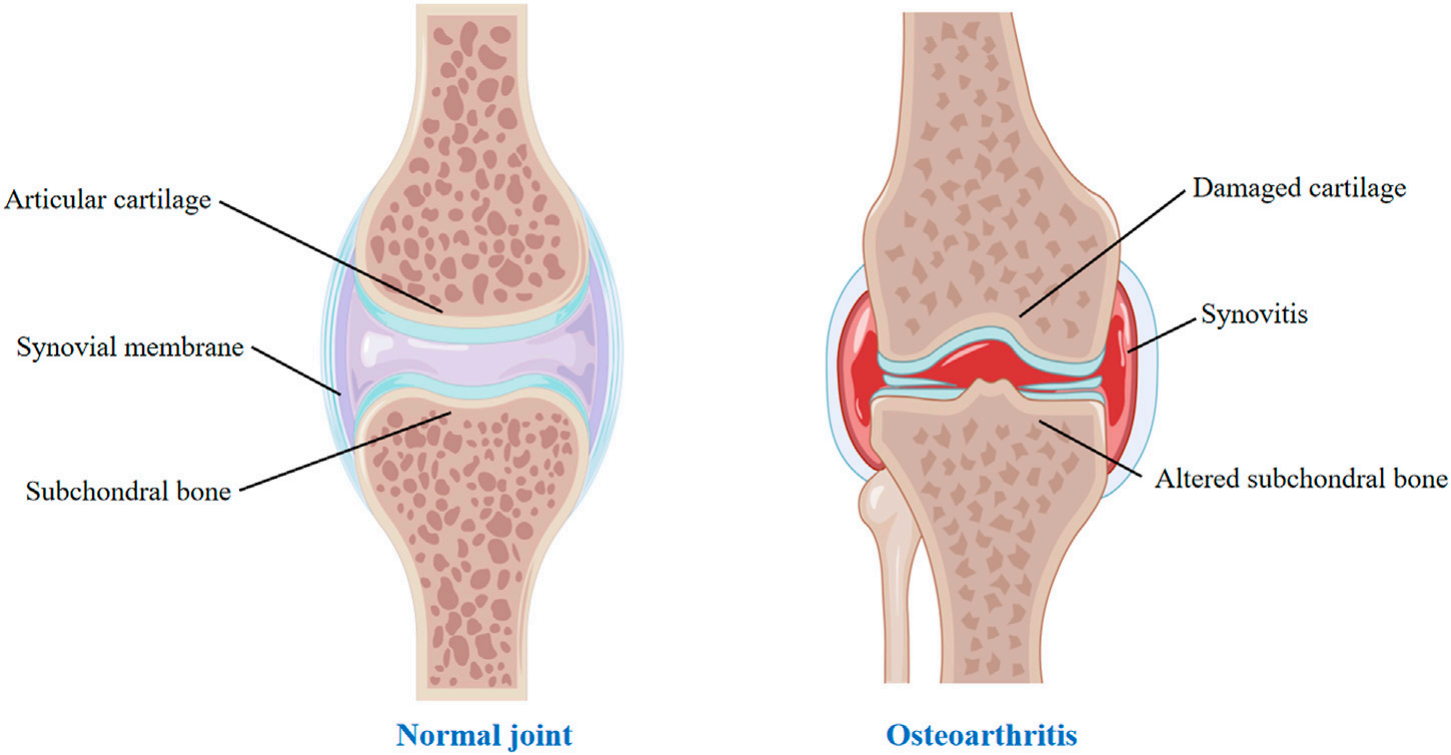
Normal joint and OA joint.

Osteoarthritis (OA), also known as degenerative osteoarthritis, is the most common form of arthritis, affecting millions of people worldwide. The primary features of OA are degenerative damage to the articular cartilage, responsive hyperplasia of the articular rim and subchondral bone (Ruiz-Romero and Blanco, 2009) (Figure 1). It occurs when the protective cartilage that cushions the ends of the bones wears down over time while increasing age is a dependent risk factor for the development of osteoarthritis (Sun et al., 2019). It is reported that there is a prevalence of 28.7% of OA all over the world (Kumar et al., 2020). OA is prone to weight-bearing joints (such as cervical spine, lumbar spine, knee joint, hip joint, etc.), and especially, the knee joint is one of the most common sites that occurs osteoarthritis. Knee Osteoarthritis is frequent in the middle-aged and elderly population, which is a leading reason that causing disability in geriatric population of society, bringing great pain to patients.

**FIGURE 2:**
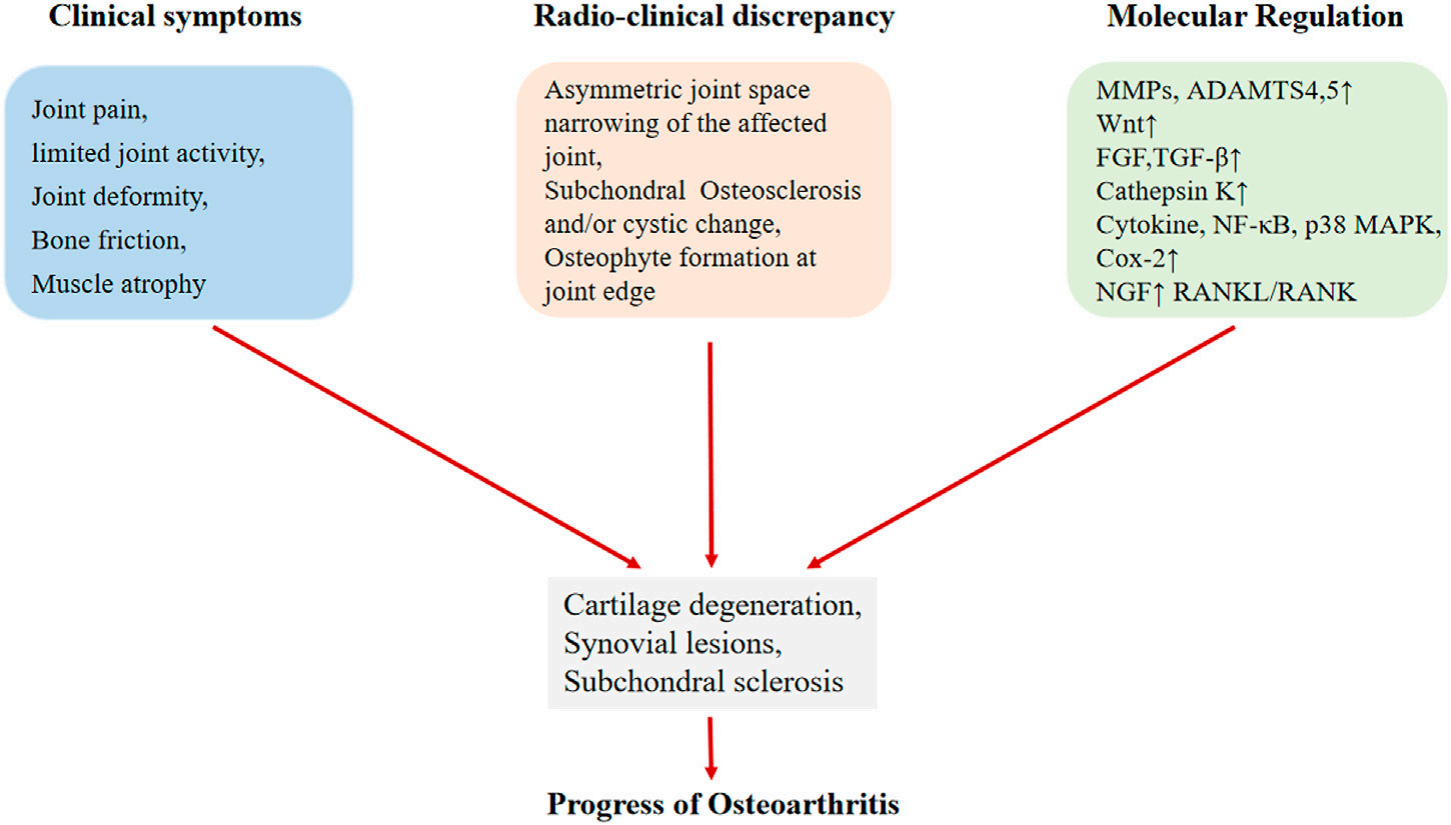
The development of osteoarthritis includes clinical symptoms, radiation clinical progress and molecular mechanism changes.

The previous studies have shown that the pathogenesis of osteoarthritis is mainly related to three key aspects: articular cartilage degeneration, synovial immune pathogenesis and subchondral bone changes (Wei and Bai, 2016). The change of subchondral bone plays an important role in the progress of OA. In the progressive stage, the subchondral bone underwent a series of changes, such as extensive remodeling of the trabecular bone, the formation of new bone below the cartilage, the development of subchondral bone cysts (SBC) and the thickening of the growth plate (Milgram, 1983; Felson, 2004; Anderson-Mackenzie et al., 2005; Thambyah and Broom, 2009) (Figure 2). In addition, animal experiments showed that subchondral bone thinning occurred before cartilage damage, indicating that there may be increased bone turnover in the early stage of OA (Klose-Jensen et al., 2015). Osteoclasts play a very important role in initiating subchondral bone remodeling in the innervation of pain sensation in osteoarthritis (Zhu et al., 2019), halting the changes in subchondral bone induced by osteoclast activation may be a potential treatment direction of osteoarthritis.

Although there are available methods and ongoing research for the clinical treatment of OA, there is still no “gold standard” for the treatment of severe osteoarthritis. In the current clinical practice, non-steroidal anti-inflammatory drugs (NSAIDs), glucocorticoids, chondroitin are the first-line treatment for different types of OA. However, they can only manage the symptoms of OA, but not reverse the damage to joints, while they are very likely to induce adverse events such as gastrointestinal tract, cardiovascular, liver and kidney (Da et al., 2021; Dahmer and Schiller, 2008; Q; Wang et al., 2022). Besides, intra-articular injection of Corticol, platelet-rich plasma or hyaluronic acid only has short-term effect. And it is not recommended the routine intra-articular injection of hyaluronic acid (Brophy and Fillingham, 2022). With the rapid development of the pathology of OA, small-molecule drugs become an emerging potential choice for OA treatment. Compared with conditional treatment, small-molecule compounds have many advantages, such as clear components, definite pharmacological effect and pharmacokinetic parameters, fewer side effects, better tolerance and so on. Most importantly, small-molecule drugs might not only relieve symptoms but also rebuild normal cartilage structure to restore joint functions, which brings hope for OA patients to completely cure the disease. This unique feature makes small-molecular drugs become a research hotspot in the field of OA treatment. However, there is a lack of a summary that can objectively analyze the current achievement and research trends in this booming field. Considering the above reasons, we reviewed the research status of small molecule compounds based on the pathogenesis of osteoarthritis, in order to provide ideas for the research and development of anti-OA drugs.

### 1.1 Small molecule inhibitors for articular cartilage

**FIGURE 3:**
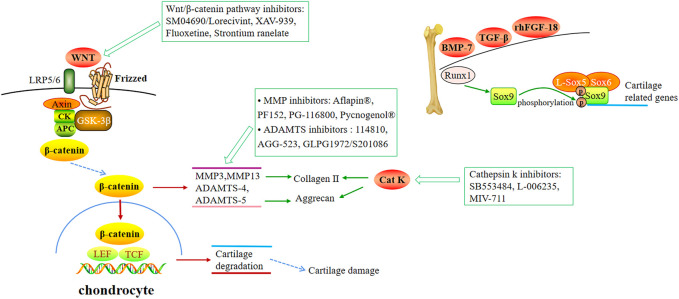
Small molecular therapeutic agents focused on targeting articular cartilage.

Cartilage degeneration is the most common cause of OA, drugs that regulate cartilage metabolism and delay cartilage degradation are shown in Figure 3.

## 2 Matrix metalloproteinases inhibitors

### 2.1 Matrix metalloproteinase (MMP) 13

MMPs are zinc dependent and effective endopeptidases, which are responsible for cutting various ECM proteins and participating in the degradation and remodeling of cartilage extracellular matrix (Hu and Ecker, 2021). Most of the 25 subtypes of MMPs, including MMP-1, MMP-2, MMP-3, MMP-8, MMP-9, MMP-10, MMP-13 and MMP-14, participate in the turnover of ECM in OA and the related destruction of articular cartilage (Rowan et al., 2008). Among them, soluble collagenase MMP-1, MMP-8 and MMP-13 are crucial for the occurrence of such damage, especially MMP-13 plays a leading role. MMP13 could degrade type II collagen in articular cartilage and bone. It is worth noting that MMP13 is specifically expressed in degenerative cartilage of OA patients, but not in normal adult cartilage (Wan et al., 2020). Therefore, the development of selective MMP13 inhibitors is a potential strategy for the treatment of OA.

Aflapin^®^, also known as AprèsFlex^®^, is capable of inhibiting MMP-3. A randomized, double-blind, placebo-controlled clinical trial confirmed that Aflapin is clinically effective, fast and safe in the treatment of osteoarthritis with no obvious adverse reactions (Karlapudi et al., 2023). PF152 can reduce the degradation of human cartilage *in vitro*, and has the ability to reduce the severity of articular cartilage damage in OA dogs caused by partial medial meniscectomy (Settle et al., 2010). However, other preclinical tests of PF152 have shown significant nephrotoxicity (Ruminski et al., 2016a). PG-116800 is a member of hydroxyproline-based hydroxamic acid MMP inhibitors. In a randomized, 12-month, double-blind, placebo-controlled study, patients with knee osteoarthritis developed musculoskeletal toxicity after taking PG-116800, but there was no obvious benefit (Krzeski et al., 2007). Pycnogenol^®^ has been shown to significantly reduce serum MMP-3 and MMP-13 in patients with severe OA after taking it for 3 weeks (100 mg twice daily) (Jessberger et al., 2017; Mulek et al., 2017). At the same time, through three double-blind randomized clinical trials, patients received 100 mg (Belcaro et al., 2008) or 150 mg (Rohdewald, 2018) every day for 3 months, which can significantly reduce the WOMAC score of pain and joint stiffness, but more research is still needed to evaluate its safety and effectiveness. A study by Naito et al. shows that a new neutralizing antibody for MMP-13 is developed by immunizing with synthetic peptide, which specifically binds to MMP-13 and has high selectivity for other MMPs (Naito et al., 2012). However, the therapeutic potential of anti MMP-13 antibody for OA has not yet been realized. Data on the role of MMP-13 inhibitors in OA treatment are limited, and further research is needed to develop highly selective drugs to avoid the side effects of non-selective MMP inhibitors. In the future, MMP-13 inhibitors may bring a breakthrough to the treatment of OA.

### 2.2 Platelet reactive protein integrin metallopeptidase (ADAMTS)-5

ADAMTS metalloproteinase is another secreted zinc endopeptidase. Its mechanism is to degrade proteoglycans, reduce the stability of chondrocytes, cause dramatic morphological changes, and destroy chondrocytes. ADAMTS-5 is a major “aggregating protein polyglycolase” found in animal (Stanton et al., 2005) and human OA articular cartilage (Mead and Apte, 2018). Therefore, ADAMTS-5 is a promising target for identifying and mitigating OA development. The synergistic effect of ADAMTS-5 inhibitor (114810) combined with hyaluronic acid hydrogel (HAX) was confirmed to promote the healing of cartilage in osteochondral defect model and prevent the progression of degenerative changes in ACL model (Chen et al., 2014). ADAMTS-5 inhibitors have been shown to reduce synovial joint damage in OA animal models. Therefore, a drug development plan for active ADAMTS-5 has been established.

AGG-523, a selective oral inhibitor of ADAMTS-4 and 5, entered the Phase I human study (NCT00454298), but was suspended for unknown reasons. GLPG1972/S201086 was evaluated in the Phase I OA clinical trial (NCT03311009) (van der Aar et al., 2022). In healthy volunteers and OA participants, after 2 weeks of daily administration, the concentration of aggrecan ARGS in serum or plasma decreased in a dose-dependent manner, indicating that GLPG1972 was bound to target ADAMTS-5. After single and multiple oral administration of GLPG 1972, the patient’s tolerance was good. The evaluation of the efficacy and safety of GLPG1972 has been further studied in the phase 2 clinical study of knee OA patients (NCT03595618).

## 3 Growth factor inhibitors

### 3.1 Fibroblast growth factor (FGF)

FGF is a known factor that has a significant impact on the response to injury and the progression of OA in cartilage. FGF-2 has the role of decomposition and anti-synthesis in human cartilage homeostasis (Zhang et al., 2020), and can effectively stimulate the expression of MMP-13, which is the main degrading enzyme of type II collagen. In the musculoskeletal system, FGF-18 is involved in the development and maturation of cartilage, and it has the function of enhancing regeneration and repair in mature cartilage (Hendesi et al., 2022).

Sprifermin (AS-902330) is a recombinant human FGF-18 protein molecule (rhFGF18) and a truncated FGF-18 protein molecule. *In vitro* studies have shown that Sprifermin can significantly induce chondrocyte proliferation and promote ECM generation, and Sprifermin can still exert this effect under continuous and intermittent administration, which is confirmed to play a role by activating the extracellular regulated protein kinase ERK pathway in chondrocytes. Sprifermin has the potential to promote cartilage growth and repair cartilage, thus delaying the progression of OA and improving the patient’s condition (Gigout et al., 2017). At present, the drug has completed the Phase I clinical trial. The results showed that 180 patients with knee osteoarthritis were significantly improved after the drug was injected into the bone joint cavity. Specifically, the decrease in the thickness of cartilage in patients was alleviated, and the thickness of cartilage in the leg tibia and both sides of the joint cavity was significantly thicker than that in the placebo group, which was dose dependent. At the same time, the patient did not have serious adverse reactions (Lohmander et al., 2014). At present, the drug is still in a 5-year multicenter, randomized, double-blind, placebo-controlled and parallel controlled phase II clinical trial (NCT01919164), and has completed 2-year data collection and analysis. Compared with the placebo group, after 2 years of treatment, the cartilage thickness of the two groups of patients receiving high-dose Sprifermin showed a significant dose-dependent increase. However, the first meta-analysis that aims to provide a comprehensive evaluation of the efficacy and safety of intra-articular sprifermin injections demonstrated that it is unlikely for intra-articular sprifermin to significant improve in physical function and clinical symptoms in knee OA patients (Zeng et al., 2021). While sprifermin can be regarded as a potential DMOAD for OA patients, more evidence is still required for its efficacy and safety.

### 3.2 Transforming growth factor (TGF)-β

TGF-β inhibits the hypertrophy and maturation of chondrocytes, and has anabolic function in the pathological participation of articular cartilage homeostasis and subchondral bone. Thus, inhibiting TGF-β signal transduction is a potential mechanism for the occurrence and development of OA.

It is reported that TGF is injected-β Type 1 receptor inhibitor (SB-505124) or alginate beads implanted into TGF-β Antibody (1D11) can alleviate the disease of OA mice/ratsinduced by anterior cruciate ligament transection (ACLT) (Zhen et al., 2013). A phase II clinical trial (NCT01221441) identified TGF expression-β1 (GEC-TGF-β 1) efficacy and safety of genetically engineered allogenic human chondrocytes in patients with grade 3 chronic knee degenerative disease (Cherian et al., 2015). The results showed that compared with the placebo group, GEC-TGF was accepted because the percentage of treated patients who receive painkillers was decreased. With transforming growth factor (TGF-β1) expression vector virus transduced genetically engineered chondrocytes have been proven to have potential benefits in the non-surgical treatment of knee osteoarthritis. A multicenter, double-blind, placebo-controlled, randomized study was conducted in adults with K-L III knee osteoarthritis. In the TG-C cohort, full knee MRI at 12 months showed less progress in cartilage damage, subpatellar fat pad synovitis, and effusion synovitis. The IKDC and VAS scores of patients receiving TG-C treatment were significantly improved. In addition, among patients receiving TG-C treatment, cartilage damage progressed less, and subpatellar fat pad synovitis and effusion synovitis progressed less. In addition, TG-C treatment is generally well tolerated, with minor adverse events (Lee et al., 2020). Therefore, TG-C seems to be a safe and effective method to treat K-L III osteoarthritis.

### 3.3 Bone morphogenetic protein (BMP-7)

Members of bone morphogenetic protein (BMP) family, especially osteogenic protein −1 (OP-1) (also known as BMP-7), have shown great potential as anabolic factors of cartilage, because they have the ability to induce matrix synthesis and promote cartilage repair, and play a key role in bone and cartilage homeostasis and regeneration (Carreira et al., 2014). A single dose incremental RCT (NCT00456157) showed that the injection of 0.1 and 0.3 mg of BMP-7 improved pain scores and increased OARSI response rate compared with placebo, while the injection of 1 mg of BMP-7 was associated with injection site pain (Hunter et al., 2010). Most adverse events in the BMP-7 group were classified as mild or moderate, similar to those in the placebo group. Further Phase II trials using the 0.1 and 0.3 mg dose groups are ongoing. The double-blind, randomized, single dose incremental safety study of bone morphogenetic protein (38A BMP-7) in knee OA subjects (NCT01133613) and the dose discovery study of BMP-7 in knee osteoarthritis patients (NCT01111045) are also being further evaluated.

## 4 Wnt/β-Catenin inhibitors

Wnt signaling pathways regulate key biological processes in development, growth, homeostasis, and disease, particularly in joints and bones. The elevated Wnt signal in chondrocytes can promote its proliferation and hypertrophy differentiation, and have harmful effects on cartilage homeostasis (Dell'Accio and Cailotto, 2018). The excessive activation of Wnt signaling pathway in articular cartilage is significantly related to the onset and severity of osteoarthritis. More and more evidences show their pathological role in OA. Therefore, inhibition of Wnt signal has been explored as a potential treatment for OA.

SM04690 (Lorecivint) is a small molecule Wnt pathway inhibitor, which is currently under development. As a potential DMOAD for the treatment of knee OA, it has developed from *in vitro* evaluation to human clinical trials. SM04690 can induce cartilage formation and inhibit joint damage in rat OA model, which is a candidate drug for relieving potential disease of OA. A 24-week phase 1 study (NCT02095548) for patients with moderate to severe knee OA showed that SM04690 was well tolerated and safe (Yazici et al., 2017). Adverse reactions possibly related to treatment were observed mainly in patients receiving high dose SM04690 (0.23 mg) (Yazici et al., 2017). A 52 weeks, multicenter, randomized, double-blind phase IIa clinical trial of SM04690 (NCT02536833) confirmed that SM04690 had clinically significant improvement in WOMAC pain and function scores in patients with moderate to severe knee OA (Yazici et al., 2020). Another Wnt/β-catenin signal inhibitor XAV-939 was found to reduce the severity of the disease in the traumatic OA model by promoting the anti-metabolism of chondrocytes and the anti-fibrosis of synovial fibroblasts (Lietman et al., 2018). Fluoxetine has been widely used since it was approved by FDA in 1986. In recent years, it has been found that fluoxetine can not only inhibit the abnormal activation of Wnt/β-catenin signal transduction in OA patients, but also relieve the pain related to osteoarthritis (Miyamoto et al., 2017). The Phase III clinical trial (ISRCTN41323372) of strontium ranelate SrR in the treatment of knee osteoarthritis (SEKIOA) provided the most reliable clinical evidence. In this 3-year multicenter randomized controlled trial (Reginster et al., 2013), compared with patients treated with placebo, patients treated with SrR showed significant pain relief, functional improvement and radiation improvement. The study found that SrR could inhibit Wnt/β-Catenin pathway promotes cartilage formation (Yu et al., 2021). Nevertheless, SrR has been discontinued due to safety issues.

## 5 Cathepsin K inhibitors

Cathepsin K is a cysteine protease, which is involved in the degradation of key components of bone and cartilage, such as type I and type II collagen, bone remodeling/absorption and articular cartilage degradation (Charni-Ben et al., 2008). Although the cathepsin K inhibitor SB553484 is less selective than other cathepsins, it plays a cartilage protection role in dogs receiving partial medial meniscectomy (Connor et al., 2009). L-006235, a selective cathepsin K inhibitor, reversed the loss of subchondral bone in rabbits with anterior cruciate ligament transection (ACLT), and subchondral bone protection was related to cartilage protection (Hayami et al., 2012). However, neither of these compounds has entered the clinical development stage. MIV-711 is a highly effective selective cathepsin K inhibitor, which is currently in the OA II phase. The results of the initial IIa study were recently reported (Bihlet et al., 2022). Compared with patients receiving placebo treatment, knee OA patients receiving MIV-711 treatment for 6 months benefit from displaying joint structure once a day, and the increase of bone area and cartilage thinning of affected knee joints is significantly reduced. A randomized phase 2a clinical trial conducted an exploratory analysis of participants in the MIV-711 trial who mainly suffered from unilateral knee pain (Bihlet et al., 2022). MIV-711 100 mg treatment can significantly reduce WOMAC pain and bone area change.

### 5.1 Small molecule inhibitor for inhibiting osteogenesis

**FIGURE 4:**
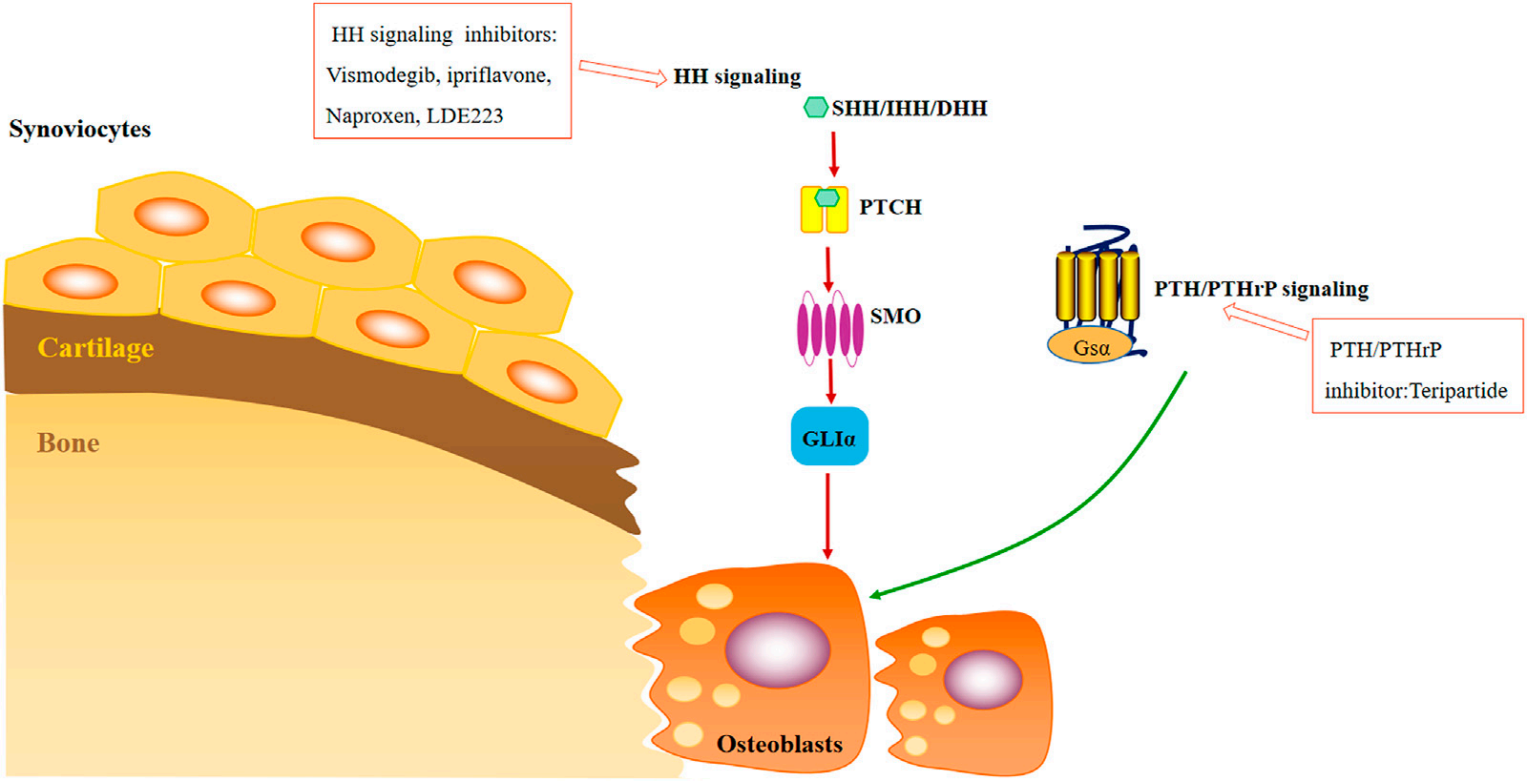
Small molecular therapeutic agents focused on inhibiting osteogenesis.

Recent observations have demonstrated that OA is associated with early loss of bone owing to increased bone remodeling (Burr and Gallant, 2012). It is reported that the abnormal upregulation of bone resorption of osteoclasts in subchondral bone leads to cartilage loss in the early stage of OA (Lin et al., 2018). Increased osteoclast activity and significant reduction in subchondral bone thickness, leading to loss of structural integrity of the osteochondral junction and promoting cartilage degeneration (Li et al., 2013). Inhibition of abnormal upregulation of bone resorption of osteoclasts is as important as cartilage repair in inhibiting the progression of osteoarthritis. Inhibition of HH pathway and PTH/PTHrP regulating axis are reported to be effective to prevent cartilage degradation. The detailed mechanism can be seen in Figure 4 (Ruiz-Heiland et al., 2012). Osteoclasts originate from hematopoietic cell lines and have good ability to degrade mineralized bone and cartilage matrix-κB ligand receptor activator (RANKL) is differentiated (Fumoto et al., 2014; Park et al., 2017). The combination of M-CSF and c-FMS maintains the survival and proliferation of bone marrow mononuclear cells (BMMCs) and osteoclasts, and initiates the differentiation of BMMCs into osteoclast precursors. While the combination of RANKL and RANK leads to the differentiation of terminal mature osteoclasts (Kim and Kim, 2016)**.**

### 5.2 Hedgehog (HH) signal pathway inhibitors

The HH signal pathway is a highly conserved cellular signal pathway. In OA, articular chondrocytes have experienced phenotypic and gene expression changes similar to those of hypertrophic growth plate chondrocytes in long bone formation. The increased HH signal has been proved to drive these changes in articular chondrocytes in adult life. Inhibiting the HH signal can prevent cartilage degradation and promote the repair of OA animal models (Ruiz-Heiland et al., 2012). During the development of human OA, the activation of HH signal promotes the expression of OA markers, such as collagen X and MMP13, for chondrocyte hypertrophy closely related to cartilage degeneration (Wei et al., 2012). These evidences indicate that HH signal is the therapeutic target of OA, and inhibiting the HH signal in articular cartilage can prevent or delay the progression of adult OA.

Vismodegib, as an exogenous HH antagonist, has been approved by FDA for clinical treatment of patients with basal cell carcinoma. Research shows that vismodegib has a potential therapeutic effect in the treatment of osteoarthritis (Chen et al., 2022). Ipriflavone, a new and safe HH signal inhibitor, reduces cartilage degradation by blocking the HH pathway and destroys catabolic genes, providing cartilage protection for post-traumatic OA in rats (Guo et al., 2019). Naproxen (Npx) can affect gene expression during osteoblastic differentiation of mesenchymal stem cells, and downregulate mineral deposition in ECM through HH signal, further affecting MSC mediated subchondral bone repair in patients with osteoarthritis (Salem et al., 2014). LDE223 inhibits SMO (a key component of hedgehog pathway) to inhibit the formation of osteophyte in the mouse osteoarthritis model. Inhibiting hedgehog signal transduction by targeting SMO can specifically inhibit the formation of osteophyte in arthritis without affecting inflammation and causing bone destruction at local and systemic levels (Ruiz-Heiland et al., 2012b).

### 5.3 Parathyroid hormone/parathyroid hormone-related protein

Parathyroid hormone (PTH) or its homologueparathyroid hormone-related protein (PTHrP) inhibits chondrocyte hypertrophy and regulates endochondral ossification in the growth plate through the IHH-PTHrP regulatory axis, and regulates subchondral bone remodeling and reduces the final differentiation of articular cartilage (Fu et al., 2019). The negative feedback loop of IHH/PTHrP is critical to chondrocyte differentiation during the formation of endochondral bone. Recombinant human PTH (1–34) terlipatide has been approved by FDA for the treatment of osteoporosis. In the established mouse model of knee osteoarthritis with meniscus/ligament injury (MLI), the ability of systematic intermittent terlipatide to slow cartilage degeneration, inhibit AC maturation and stimulate matrix synthesis was tested (Sampson et al., 2011). Although most PTH/PTHrP studies are in the preclinical stage, it is still expected to play a role in the future clinical application. A prospective phase II RCT (NCT03072147) study is currently recruiting participants to evaluate the efficacy of teripatide as a cartilage regeneration therapy for osteoarthritis.

### 5.4 RANKL-mediated signal pathway active compounds

**FIGURE 5:**
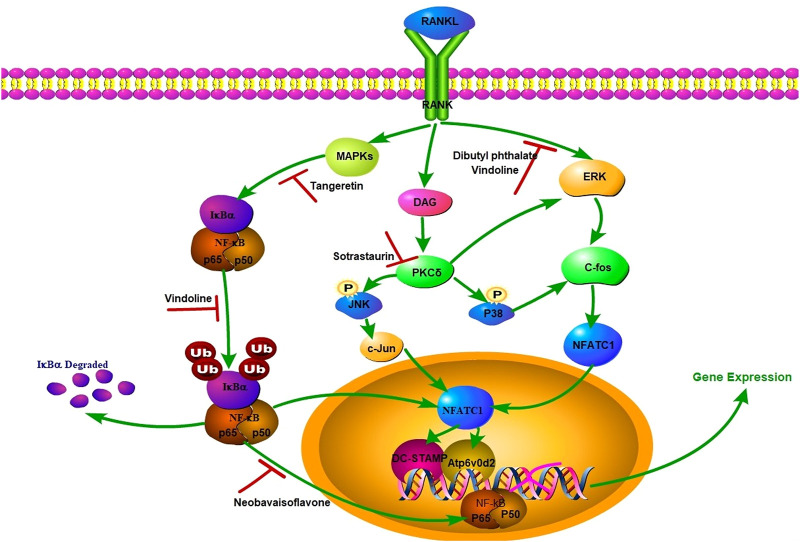
RANKL-mediated signaling pathway and natural small molecular compounds with anti-OA activity.

NF-κB and ERK signaling pathways are important in cartilage degeneration (Abu-Amer, 2012; C; Li et al., 2019). RANKL starts the activation of extracellular signal-regulated kinase (ERK), and then induces the activation of c-fos and activated T nuclear factor cytoplasmic 1 (NFATc1), which are two basic transcription factors for osteoclast fusion (Monje et al., 2005; Ma et al., 2019; Meisong Zhu et al., 2021). NFATc1 migrates into the nucleus and combines with the promoter sequence of DC-STAMP and Atp6v0d2, which are two central regulators of osteoclast fusion. At the same time, RANKL can also start NF-κB pathway to achieve osteoclast fusion (Ma et al., 2019; Meisong Zhu et al., 2021). In NF-κB pathway, iκB α attaches to NF-κB protein including p65 subunit, inhibits NF-κB phosphorylation and then migrates to the nucleus. RANKL can induce p65 phosphorylation and iκB α degradation, and NFATc1 activates and migrates to the nucleus to activate specific genes in combination with specific DNA sites. Such as tartrate-resistant acid phosphatase (TRAP), DC-STAMP, calcitonin receptor and V-ATPase d2, all of which will affect the differentiation of osteoclast precursors into mature osteoclasts (Abu-Amer, 2012; Ouyang et al., 2014; Meisong Zhu et al., 2021). MMP13, ADAMTS4 and ADAMTS5 are key regulators of cartilage destruction, and SOX9 is the main regulator of cartilage formation, which can effectively bind to single or double high mobility group (HMG)-box sites in DNA and trans-activate its target genes, such as COL2a1 and aggrecan, which participate in the formation of ECM (Kapoor et al., 2011; Song and Park, 2020). The specific mechanism is shown in Figure 5.

Panax notoginseng has been used to treat bone diseases for a long time, and has also played a potential therapeutic role in preventing OA progression and relieving pain. Fang et al. extracted dibutyl phthalate (DP) from Panax notoginseng, which can effectively inhibit the excessive activation of osteoclasts in the early stage of OA, thus preventing the rapid development of OA (Fang et al., 2022). It’s reported that DP activated osteoclast fusion by inhibiting ERK/c-fos/NFATc1 signaling pathway mediated by RANKL, thus blocking osteoclast production and preventing OA progression. In addition, osteoblasts secrete netrin-1, which induces CGRP sensory innervation in subchondral bone, and abnormal CGRP sensory innervation leads to nociceptive hyperalgesia and OA pain (Chang et al., 2005). Moreover, DP can alleviate OA pain induced by osteoclasts by inhibiting the introduction of subchondral bone into blood vessels and sensory nerves (Park et al., 2009; Zhu et al., 2019).

Vindoline (Vin) is an indole alkaloid extracted from medicinal plant Catharanthus roseus, which has anti-inflammatory properties (Fu et al., 2017). The results show that Vin can inhibit the progression of osteoarthritis by inhibiting activation of NF-κB and ERK signaling pathways induced by RANKL (M. Zhu et al., 2021). At the same time, Vin can inhibit the mRNA expression of MMP13 induced by IL-1β, upregulate the mRNA expression of COL2a1, SOX9 and aggrecan, without affecting the gene expression of ADAMTS4 and ADAMTS5, thus maintaining the steady state of ECM. The *in vivo* study showed that the number of TRAP-positive multinucleated cells in subchondral bone decreased significantly after Vin treatment.

Tangeretin is a multi-methoxy flavonoid rich in citrus peel, and it has good antioxidant pharmacological activity (Fu et al., 2017). Omar, H.A., et al. reported that hesperidin can inhibit MAPK signaling pathway and alleviate cisplatin-induced liver injury (Omar et al., 2016). IL-1β upregulates the production of iNOS and COX2, and then promotes the expression of NO and PEG2. These processes induce ECM catabolism and promote OA development by up-regulating the expression of MMPs and ADAMTS and inhibiting the synthesis of collagen II and proteoglycan (Wang and He, 2018). Studies have shown that hesperidin can inhibit the degradation of aggrecan and collagen II protein mediated by IL-1β. Simultaneously, the progression OA can be prevented by inhibiting the secretion of inflammatory mediators and ECM degradation of chondrocytes and ROS production in OA by activating Nrf2/HO-1 axis. In addition, Tangeretin can also downregulate the activation of NF-κB by inhibiting MAPK signaling pathway.

Neobavaisoflavone (NBIF) is an isoflavone compound, which was originally isolated from *Arabidopsis thaliana* seeds. Because of its anti-inflammatory, anti-cancer and anti-oxidation properties, it has attracted wide attention (Goldring and Goldring, 2010; Chen et al., 2020). Researchers found that NBIF may have potential therapeutic effect on OA through 2D cell membrane chromatography/C18 column/time of flight mass spectrometry (2D CMC/C18 column/TOFMS) system and reported the effect of NBIF on osteoclast differentiation (Tu, 1999). It is reported that NBIF intervention can significantly weaken the release of Ca^2+^ from stimulated cytoplasm induced by RANKL. Ca2+ oscillation is caused by AKT signal activation and is an important messenger of osteoclast differentiation signal transduction, so NBIF may also organize osteoclast differentiation by blocking Akt pathway stimulated by RANKL.

Dihydroartemisinin (DHA) is an effective antimalarial drug and a semi-synthetic derivative of artemisinin. It plays an important role in inhibiting infection, angiogenesis, cancer and osteoclast formation (Honma et al., 2014; Cui et al., 2016; Feng et al., 2016). DHA can reducing the inhibitory effect on sclerosing and alleviating the abnormal remodeling of subchondral bone by reducing the expression of osteoclast-derived factor (LIF). LIF is a bone remodeling regulatory protein secreted by osteoclasts, which binds to the receptor complex of glycoprotein 130 (GP130)/LIFR on bone cells and has the effect of inhibiting sclerosing (Y. Li et al., 2019; Ma et al., 2021). DHA can slow down the abnormal remodeling of subchondral bone by inhibiting the abnormal angiogenesis of subchondral bone (Pfeifhofer et al., 2006). In addition, Skvara et al. proved that Artemisia annua succinyl can reduce articular cartilage degeneration by inhibiting abnormal bone remodeling and angiogenesis (Skvara et al., 2008).

Sotrastaurin (SO) is a small molecular weight indolyl maleimide immunosuppressant, which can selectively inhibit PKC (Cremasco et al., 2012; Pang et al., 2020). Recent studies have shown that the protein kinase C family of serine/threonine kinases, especially the PKC highly expressed in osteoclasts, it also plays an important regulatory role in osteoclast formation and bone absorption (Khor et al., 2013). PKCδ deficiency attenuates bone resorption activity of mature osteoclasts *in vivo* and *in vitro* by affecting the formation of fold edges and inhibiting the secretion of histone K, a key protease that destroys the mineralized bone and cartilage matrix (Kc et al., 2016). In addition, PKC δ may upregulation of MAPK pathway (Kc et al., 2016). Pang et al. also showed that the anti-osteoclast effect of SO was partly due to the reduction of RANKL-induced MAPK signal pathway and the downstream induction of nuclear transcription factors c-fos, c-jun and nfatc1, thus down-regulating the expression of key genes required for the differentiation and fusion of precursor cells and the bone absorption of mature osteoclasts (Pang et al., 2020).

### 5.5 Small molecule inhibitor for controlling inflammation

**FIGURE 6:**
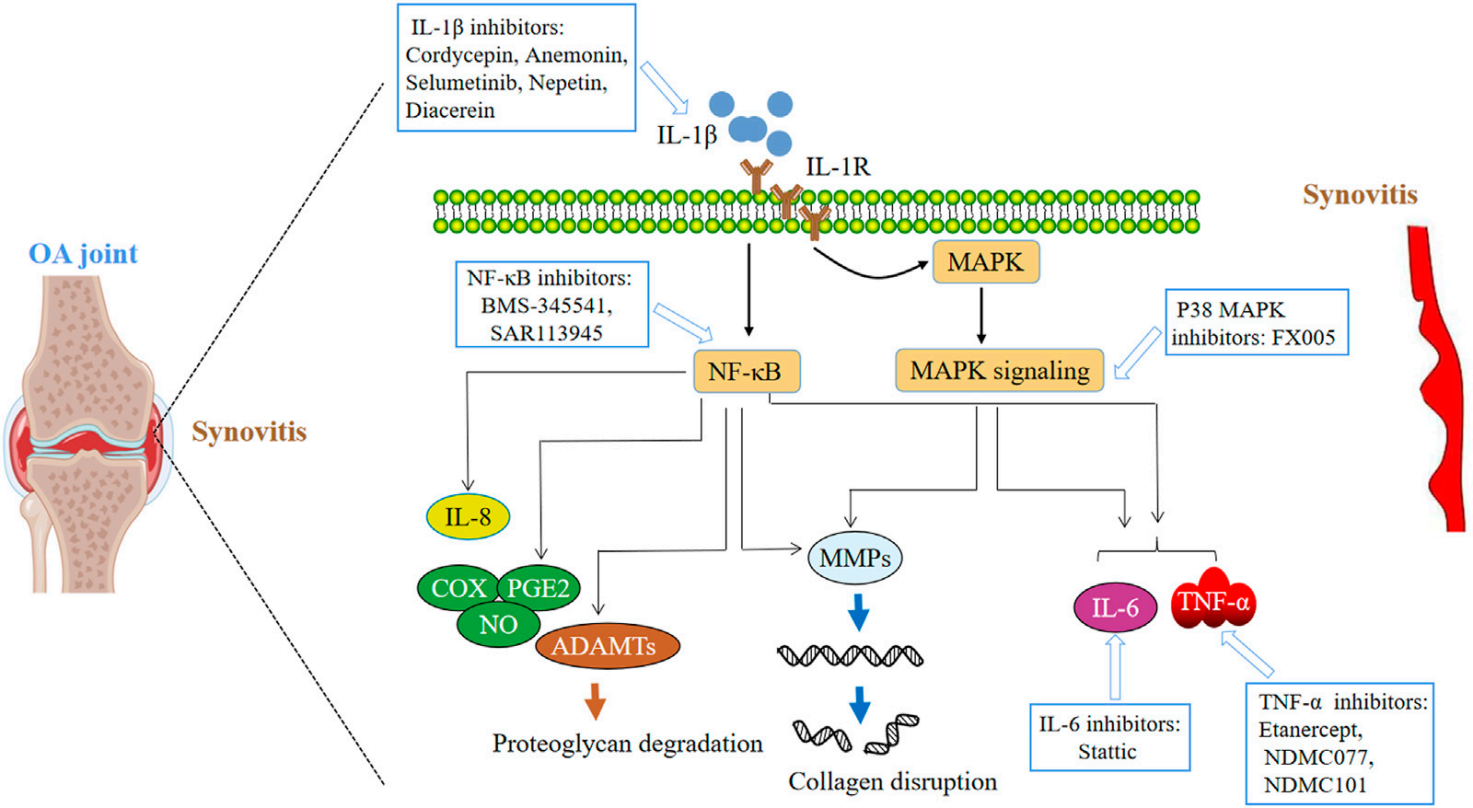
Small molecular therapeutic agents focused on inflammatory mediators.

Local and systemic inflammation plays a key role in the pathogenesis and progression of OA, and anti-inflammatory therapy has a research prospect in reducing the progression of OA (Figure 6).

## 6 Cytokines and small molecule inhibitors

TNF-α can promote the expression of some matrix metalloproteinases and accelerate the destruction of ECM. At the same time, the synthesis of a variety of pathogenic factors (such as prostaglandins) increases, accelerating the occurrence of OA (Lai et al., 2014). TNF-α inhibitors may be a potential choice for the treatment of OA. TNF-α inhibitor Etanercept can reduce the level of serum MMP-3, but it has no effect on pain improvement in patients with inflammatory hand osteoarthritis (Kraus et al., 2012; Kloppenburg et al., 2018; Kroon et al., 2020). By using TNF-α-induced chondrocyte inflammatory reaction as a screening tool, two effective amide-linked small molecules (HS Cf and HS Cm) were identified from a small chemical library. HS Cf (NDMC077) has a protective effect on cartilage, and can prevent the cartilage destruction induced by TNF-α by down-regulating the signal of interferon regulatory factor-1(IRF-1) (Liu et al., 2011). HS Cm (NDMC101) is an effective immunomodulator with anti-inflammatory activity in inflammation-related diseases (Liou et al., 2014). All these results are encouraging. Anti-inflammatory drugs can prevent cartilage damage induced by TNF-α in osteoarthritis.

The expression of IL-1 in OA patients was positively correlated with the degree of joint swelling and pain, decreased motor function and progressive narrowing of joint space. IL-6 also plays a prominent role in OA patients. This factor promotes the imbalance of local tissue immune status, leading to the destruction of chondrocytes and the loss of compensatory ability. Cordycepin is a nucleoside derivative isolated from Cordyceps. Pretreatment with Cordyceps sinensis significantly inhibited the production of PGE2 and NO induced by IL-1β. In addition, the gene expression stimulated by IL-1β and the production of MMP-13, IL-6, iNOS and COX-2 in OA chondrocytes were significantly decreased, and the activation of NF-κB induced by IL-1β was weakened (Ying et al., 2014). This study showed the anti-inflammatory activity of cordycepin in human OA chondrocytes. Anemonin (ANE) can attenuate the articular cartilage degeneration in murine DMM model and human cartilage explants in part by inhibiting the activation of IL-1β/NF-κB pathway (Wang et al., 2017). Selumetinib could reduce cartilage inflammation caused by IL-1β. In addition, Selumetinib inhibits MAPK and NF- κB signal pathway plays a specific protective role (Zheng et al., 2022). In chondrocytes, nepetin treatment inhibits IL-1β Induced overexpression of pro-inflammatory cytokines and mediators. In addition, nepetin showed protective and therapeutic effects on mouse OA model (Zheng et al., 2022). This study inidcated that nepetin has potentials to be a new therapeutic option in OA. Diacetoreine is a small molecule of IL-1β Inhibitors. In a 3-year randomized controlled trial, 507 patients with hip OA received daily treatment with diacerin or placebo. Although pain and dysfunction related to OA remained unchanged, diaceretin significantly reduced joint space stenosis compared with placebo (Dougados et al., 2001). IL-6 mainly induces chondrocyte catabolism through STAT-3 signal transduction, which is activated in articular cartilage affected by medial meniscus OA. Systemic blocking of IL-6 or STAT-3 by neutralizing antibody or small molecule Stattic alleviates the need for surgery in the medial meniscus induced OA model (Latourte et al., 2017).

## 7 NF-κb inhibitors

NF-κB is abnormally activated in OA and participates in many OA related events, including chondrocyte catabolism, chondrocyte survival and synovial inflammation. Therefore, NF- κB signals, including synergistic factors and downstream effector factors, are considered as potential targets of OA treatment intervention. In mouse knee OA model, intra-articular administration of certain concentration of IKK inhibitor BMS-345541 may reduce NF-κB and HIF-2α. The signal axis inhibited the development of OA in mouse knee joint. This inhibition is also replicated in human cells *in vitro* (Murahashi et al., 2018). SAR113945 is an IκB kinase inhibitor. Three phase I studies have confirmed the safety and tolerability of SAR113945. In these studies, SAR113945 shows a positive trend in WOMAC score. Phase 2a study failed to show any effect on WOMAC pain score in the overall group of recruited study participants, but showed statistically significant difference in the subgroup of patients with effusion at baseline (Grothe et al., 2017).

## 8 P38 MAPK pathway inhibitors

P38 signaling pathway plays a key role in the progress of many human diseases, especially in the development of OA (Sun et al., 2017). The activation of p38 MAPK signaling pathway may lead to the expression of proinflammatory cytokines, chemokines, MMPs and signal enzymes in human osteoarthritis chondrocytes. P38 inhibitors can inhibit chondrocyte apoptosis, reduce the production of downstream inflammatory cytokines, and prevent the recruitment of other inflammatory cells that may lead to bone and cartilage degradation (Sun et al., 2017). FX005 is a polylactic acid co glycolic acid microsphere that sustains the release of p38 creatine kinase inhibitor molecule. The efficacy and safety of this preparation were evaluated in the clinical trial (NCT01291914). The results showed that FX005 significantly alleviated pain than placebo.

### 8.1 Small molecule inhibitors for pain relief

**FIGURE 7:**
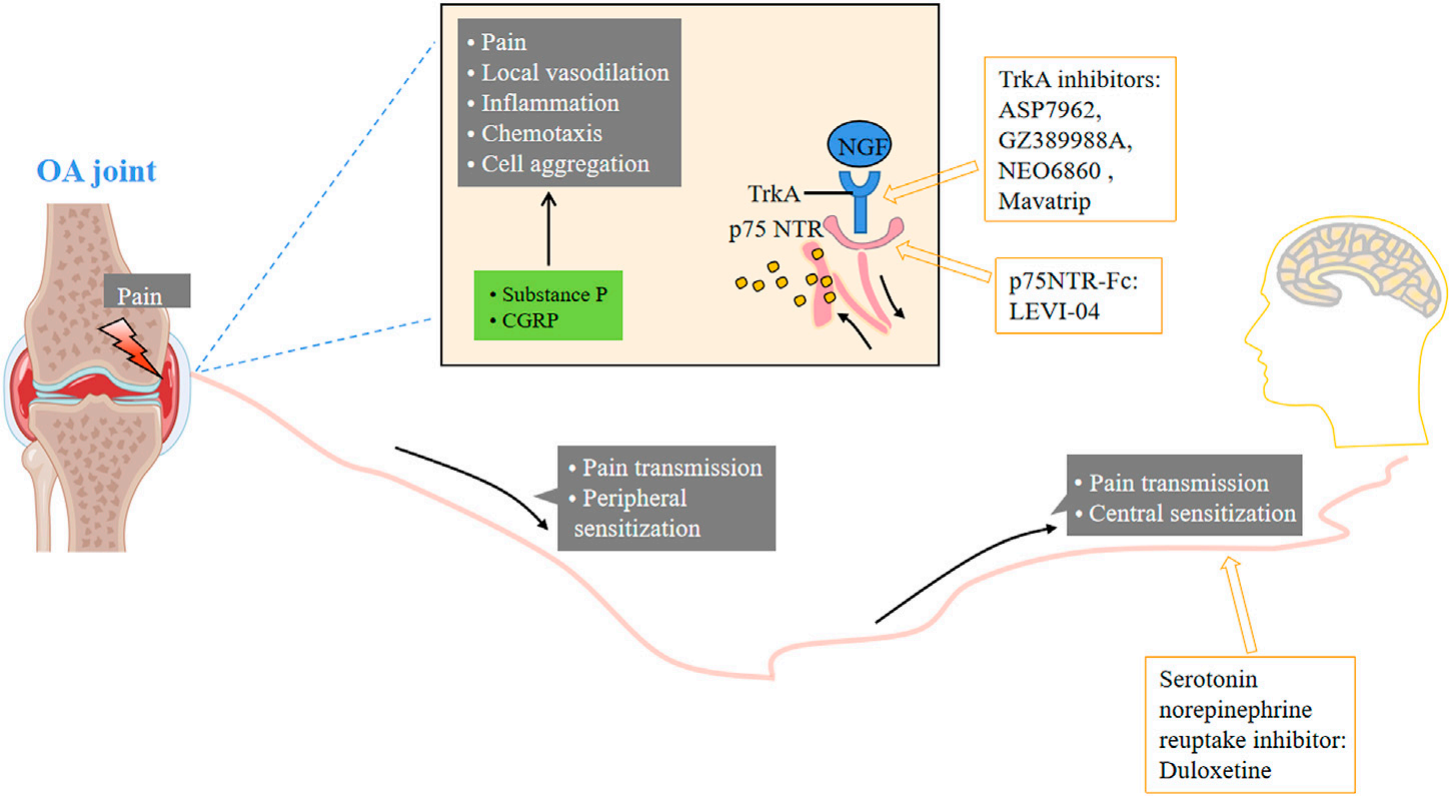
Small molecular therapeutic agents focused on pain. NGF: nerve growth factor; TrkA: tropomyosin kinase A.

Pain is also one of the important symptoms of OA, which seriously affects the quality of life of patients. It is necessary to effectively control OA pain (Figure 7).

## 9 Nerve growth factor (NGF) inhibitors

NGF is a neurotrophic factor associated with pain signal transduction and nociceptor receptor gene expression. It is a nociceptor sensitizer and has been studied as a potential target for the treatment of OA pain. It is reported that the level of NGF in synovial fluid of OA patients is significantly higher than that of normal subjects (Bain and Brazil, 2003; Baldini et al., 2020). NGF is also considered as an important pathogenic factor or pathological product in the pain process. Inhibiting the binding of NGF and its receptor can downregulate the expression of NGF, thereby reducing pain, improving limb function and relieving OA symptoms. NGF and its receptor TrkA, as possible targets for the treatment of OA pain, have been widely concerned, especially after it was reported that the pain of patients with moderate and severe knee OA was significantly relieved (Lane et al., 2010).

The new strategy to inhibit NGF induced pain focuses on the antagonism of its receptor tropomyosin related kinase A (TrkA) and p75 NTR. Blocking TrkA or p75 NTR is another strategy for suppressing NGF downstream signals, which may have less adverse effects. A randomized, double-blind, 3-cycle crossover phase II study compared 54 patients with knee osteoarthritis who took TRPV1 antagonist NEO6860 (500 mg, twice daily), placebo and naproxen for 1 day (2 doses). In this exploratory study, NEO6860 was not statistically superior to placebo, but showed a trend of analgesia (Arsenault et al., 2018). In a double-blind, randomized, placebo-controlled phase 1 study, the pharmacokinetics and pharmacodynamics of the TRPV1 receptor antagonist mavatrip in healthy men and patients with knee osteoarthritis were evaluated (Manitpisitkul et al., 2018). Compared with the placebo group, the pain intensity at rest on the 22nd day and the pain intensity after climbing stairs in the 25 mg and 50 mg dose groups were significantly lower than the baseline. The 50 mg group also showed significant pain relief after climbing stairs on the 8th day ***.*** In the phase 2a clinical trial (NCT02611466), compared with naproxen, oral administration of the TrkA inhibitor ASP7962 did not improve the pain or physical function of patients with painful knee arthritis (Watt et al., 2019). However, intra-articular injection of the TrkA inhibitor GZ389988A significantly improved WOMAC pain and overall knee pain (NCT02845271) (Krupka et al., 2019). NGF receptor p75 NTR concentration in blood, synovial fluid and tissue macrophages of OA patients increased. LEVI-04 is a p75 neurotrophic protein receptor fusion protein (p75NTR-Fc), which is currently being studied in phase I clinical trials (NCT03227796), but no results have been obtained.

## 10 Serotonin norepinephrine reuptake inhibitors

Studies have shown that central sensitization is an important factor in mediating pain in osteoarthritis. When there is continuous harmful input, such as in osteoarthritis, these same CNS processes can increase or amplify pain. Central pain symptoms respond best to CNS neuromodulators such as serotonin norepinephrine reuptake inhibitors (SNRIs) and anticonvulsants (Clauw and Hassett, 2017). Noradrenaline can regulate the nociceptive process in the spinal cord and periaqueductal gray matter, and is a potential target to improve OA pain. A phase III randomized study aimed at the exploratory post event analysis of the efficacy of duloxetine in the treatment of knee joint pain caused by OA in patients with multiple parts of pain (Itoh et al., 2021). It was evaluated that 111 patients were treated with duloxetine for 8 weeks. Compared with conventional nursing, the addition of duloxetine seems to be beneficial to end-stage knee joint OA patients with God like symptoms (with CS risk). However, the drug seems to have no effect on patients with advanced hip OA.

## 11 Conclusions and future prospects

Since the current oral drugs and local treatment of OA has limited efficacy, the prevalence of OA makes it require simple but robust therapeutic intervention. With the rapid progress in pathology of OA, the emerging small-molecule drugs have more obvious effects and fewer side effects than traditional drugs in various manners. Firstly, small-molecule drugs generally have clear components. The definite molecular formula enables it have clearly detectable pharmacodynamic and pharmacokinetic parameters. Secondly, small-molecule drugs usually have clear intracellular or extracellular targets and efficacy, including enhancing cartilage repair, inhibiting joint degeneration, reducing inflammation and relieving pain. Some small molecule anti-OA drugs have shown promise in promoting cartilage differentiation of stem cells and reconstruction of cartilage matrix, which would bring hopes of complete relieve to OA patients. Thirdly, some small-molecule drugs are easier to be structurally modified to form their derivatives, achieving better pharmacodynamics and pharmacokinetics.

Despite the encouraging advantages, the clinical applications of small-molecule drugs for OA are still in their infancy stage. The following challenges should be well addressed.

1) Most of the small molecular active compounds initially screened have relatively extensive pharmacological actions and the disadvantages of poor activity. 2D CMC/C18 column/TOFMS system can effectively screen the target small molecules with good activity. The molecular docking technology can optimize the activity of small molecular compounds and improve their pharmacological actions, so that the possibility of developing newly discovered small molecular compounds into clinically available therapeutic drugs is greatly improved.
2) Due to the limitations of poor stability of small molecules and difficulty in targeted delivery, there are few small molecules applied in OA clinic at present. To improve the defects, nanocarrier system is introduced to achieve better targeted delivery, effective treatment and improved stability. Nanocarriers have the potential to protect active molecules during drug delivery, enhance the bioavailability of molecules in disease sites, and control the release of molecules very well (Kc et al., 2016). In addition, with the availability of crystal structures of many target proteins and the progress of computer-aided drug design technology, it seems more promising for the prospect of effective small molecules for OA treatment, e.g., compounds with the improved specificity, ideal pharmacodynamics and pharmacokinetics, and acceptable toxicological characteristics.
3) As OA is a highly heterogeneous disease, single treatment for a single joint tissue would not be effective. Thus, the combination of multi-channel targeted small molecules may achieve better therapeutic effect. For example, inflammatory pain endotype, which can benefit from a drug combination that simultaneously solves pain and inflammation. Besides, the synergistic effect of small-molecule drugs and other therapeutic strategies is also worthy of further investigation, such as immunotherapy, cell therapy and gene therapy. For instance, mesenchymal stem cells have therapeutic effect on osteoarthritis through cytokine secretion and stem cell differentiation while small-molecule drugs are expected to enhance this therapeutic effect through signal pathway regulation of stem cells. How to utilize these synergistic effects to achieve ideal therapeutic efficacy needs further consideration.
4) Transdermal drug delivery system plays a very important role in drug delivery, which has the characteristics of rapid local action, small side effects and good patient compliance (Liu et al., 2022). Some small molecular compounds have a wide range of pharmacological actions, and combined with transdermal drug delivery system can achieve the effect of rapid local administration and effective reduction of drug side effects. Such as microneedle (MNs) transdermal delivery method (J. Wang et al., 2022), which physically penetrates the skin and is more effective in delivering drugs. In addition, MN can carry small molecular drugs to provide additional options for the treatment of OA. MNs can be delivered not only in a controlled release manner but also in a targeted manner. These techniques are beneficial to the transdermal drug delivery process in arthritis, reducing the number of drug delivery and making it more convenient for patients to use. They also keep a stable drug concentration in the specific tissue to extend the efficacy duration (Aghabegi et al., 2016). There are a lot of drugs that cannot be clinical translated because of severe adverse reactions. Most of them are systemic administration, such as Strontium, which shows a significant effect on pain relief and functional improvement. Transdermal drug delivery may help to solve this problem.

In conclusion, small-molecule drugs of OA are attractive frontier that deserve further exploration. We hope this review may provide a better understanding of the advantages and disadvantages of small molecule OA targeted drugs, which will help to explore more effective clinical treatment strategies for OA.
